# Genome-wide association analysis identifies multiple loci associated with kidney disease-related traits in Korean populations

**DOI:** 10.1371/journal.pone.0194044

**Published:** 2018-03-20

**Authors:** Jeonghwan Lee, Young Lee, Boram Park, Sungho Won, Jin Suk Han, Nam Ju Heo

**Affiliations:** 1 Department of Internal Medicine, Hallym University Hangang Sacred Heart Hospital, Seoul, Korea; 2 Veterans Medical Research Institute, Veterans Health Service Medical Center, Seoul, Korea; 3 Department of Public Health Science, Seoul National University, Seoul, Korea; 4 Interdisciplinary Program of Bioinformatics, Seoul National University, Seoul, Korea; 5 Institute of Health and Environment, Seoul National University, Seoul, Korea; 6 Department of Internal Medicine, Seoul National University College of Medicine, Seoul, Korea; 7 Division of Nephrology, Department of Internal Medicine, Healthcare System Gangnam Center, Seoul National University Hospital, Seoul, Korea; Shanghai Diabetes Institute, CHINA

## Abstract

Chronic kidney disease (CKD) is an important social health problem characterized by a decrease in the kidney glomerular filtration rate (GFR). In this study, we analyzed genome-wide association studies for kidney disease-related traits using data from a Korean adult health screening cohort comprising 7,064 participants. Kidney disease-related traits analyzed include blood urea nitrogen (BUN), serum creatinine, estimated GFR, and uric acid levels. We detected two genetic loci (*SLC14A2* and an intergenic region) and 8 single nucleotide polymorphisms (SNPs) associated with BUN, 3 genetic loci (*BCAS3*, *C17orf82*, *ALDH2*) and 6 SNPs associated with serum creatinine, 3 genetic loci (*BCAS3*, *C17orf82/TBX2*, *LRP2*) and 7 SNPs associated with GFR, and 14 genetic loci (3 in *ABCG2/PKD2*, 2 in *SLC2A9*, 3 in intergenic regions on chromosome 4; *OTUB1*, *NRXN2/SLC22A12*, *CDC42BPG*, *RPS6KA4*, *SLC22A9*, and *MAP4K2* on chromosome 11) and 84 SNPs associated with uric acid levels. By comparing significant genetic loci associated with serum creatinine levels and GFR, rs9895661 in *BCAS3* and rs757608 in *C17orf82* were simultaneously associated with both traits. The SNPs rs11710227 in intergenic regions on chromosome 3 showing significant association with BUN is newly discovered. Genetic variations of multiple gene loci are associated with kidney disease-related traits, and differences in associations between kidney disease-related traits and genetic variation are dependent on the population. The meanings of the mutations identified in this study will need to be reaffirmed in other population groups in the future.

## Introduction

Chronic kidney disease (CKD) is an important health problem that increases the incidence of cardiovascular disease and overall mortality [1]. Kidneys function is usually expressed as the glomerular filtration rate (GFR) and is generally deteriorated when the GFR is decreased. Traditionally, blood urea nitrogen (BUN) and serum creatinine levels have been used as surrogate markers of kidney function deterioration. BUN reflects the amount of nitrogen in the blood and is produced as a waste product of protein metabolism [2,3]. Serum creatinine is a representative biochemical indicator of kidney function and is produced by the breakdown of muscle creatine phosphate. Because the exacerbation of kidney function and the increase in serum creatinine level are not directly proportional, the GFR is estimated using demographic and biochemical factors, such as serum creatinine levels, age and sex. Uric acid is the last product of purine metabolism, and hyperuricemia develops when renal function is reduced and uric acid excretion is decreased [4]. Hyperuricemia is prevalent in patients with kidney disease, and genetic susceptibility plays an important role in the development of hyperuricemia [5]. Risk factors for CKD and end-stage renal disease requiring dialysis are diabetes mellitus, hypertension, glomerulonephritis, and polycystic kidney disease. However, these traditional risk factors alone cannot completely explain the development of CKD [6]. Genetic studies have shown that genetic factors affect approximately 36–75% of kidney function and vulnerability to CKD progression [7,8]. Genome-wide association studies have been used to identify genetic variations associated with kidney function in various populations, and differential genetic variation was found in each population group [9,10]. In this study, we conducted a genome-wide association and replication study of a Korean adult population to identify multiple genetic loci associated with kidney disease-related traits, including, BUN, serum creatinine, GFR, and uric acid levels.

## Methods

### Study participants

Between January 2014 and December 2014, 7,999 adults who underwent a health screening assessment at the Seoul National University Hospital Health Care System Gangnam Center were asked to consent to research, and their blood samples were collected and stored for further study. Most patients voluntarily conducted a personal health check-up or submitted to a health check-up with financial support from the company. The Institutional Review Board of Seoul National University Hospital approved the storage of blood samples for genetic analysis with informed consent. To investigate associations between genetic variations and kidney disease-related traits, we retrospectively enrolled 7,064 healthy participants after performing quality control analysis on the genetic samples. All participants had undergone blood sampling to measure BUN, serum creatinine, and uric acid levels. Demographic and other clinical information (age, sex, body mass index (BMI), comorbidities, and laboratory findings) regarding the participants were collected through electronic medical record review at the time of the health check-up. The Institutional Review Board of Seoul National University Hospital approved the research plan (IRB 1603-120-750), and the study was conducted in compliance with the Helsinki Declaration. Specifically, personal information was encrypted for confidentiality, and genetic information was analyzed by professional analysts in a third space separate from personal information.

### Definition of kidney disease-related traits

The kidney function-related traits assessed in this study included BUN, serum creatinine, estimated GFR, and uric acid levels. GFRs were calculated using the Modification of Diet in Renal Disease (MDRD) estimated GFR equation.

### Genotyping

Genomic DNA was extracted from venous blood samples genotyped using Affymetrix Axiom® Customized Biobank Genotyping Arrays (Affymetrix, Santa Clara, CA, USA), and the PLINK program (ver. 1.07) was used for quality control procedures. Specimens with the following characteristics were excluded from the analysis: low genotyping call rate (≤ 97%), sex inconsistency, and related and cryptically related individuals (identical by descent > 0.9). Small nucleotide polymorphisms (SNPs) with low call rates (< 97%), low minor allele frequency (MAF ≤ 0.05%), or significant deviation from the Hardy-Weinberg equilibrium permutation test (HWE P < 1.0 × 10^−5^) were excluded (S1 Table). After performing the quality control evaluations, 345,072 autosomal SNPs were retained for the association analysis. S2–S5 Tables summarize the HWE, MAF, and missing rates of each SNP. Targeted imputation was performed in the validation set when SNP information could not be confirmed as significant in the discovery set. Imputation was performed as follows: genotypes were pre-phased using SHAPEIT2, imputed with IMPUTE2, and analyzed using 1000 genome phase 3 haplotypes as the reference panel.

### Statistical analysis

SNPs associated with kidney disease-related outcomes, including BUN serum creatinine, GFR, and uric acid levels, were identified with multiple linear or logistical regression methods with adjustments for age, sex, diabetes mellitus, hypertension, and BMI effects. Principal component (PC) scores were estimated with the EIGENSTRAT approach to adjust the population substructure, and the first five PC scores were also included as covariates [11]. A total of 345,072 SNPs that passed the quality control assessment were used for the genome-wide association study. Distributions of normality for BUN, serum creatinine, GFR, and uric acid levels were evaluated with a histogram and Kolmogorov-Smirnov test. Because the continuous variables did not show normal distribution, the characteristics of the continuous variables were displayed using median values and interquartile ranges. Categorical variables were expressed as frequencies or percentages. The R software package (version 3.1.1., R development Core Team; R Foundation for Statistical Computing, Vienna, Austria) was used for statistical analysis and to draw the Manhattan–log10 plots. Analysis results were verified using discovery and validation sets. The discovery set comprised 7,064 of the participants included in this study. Significant SNPs (P < 1.45 × 10^−7^, value derived from the 345,072 QC-qualified SNPs and Bonferroni correction) were tested in the validation cohort samples. BUN, serum creatinine, and GFR validation was performed using results from participants of the genome-wide association study of the Korea Association Resource (KARE), and uric acid validation was performed using results from participants of the Health Examinee shared control (HEXA) study. The KARE is a prospective cohort designed to identify risk factors for major chronic diseases in Koreans, including diabetes and hypertension [12–14]. The KARE cohort consists of 10,038 adults (aged 40–69) who are representative samples of residents in two cities (Ansung and Ansan) in South Korea. Data obtained from physical examinations and laboratory tests have been collected since 2001, and follow-up studies have been conducted every two years. Genetic testing results from 6,509 participants in the third phase of the KARE cohort study were used to validate BUN, serum creatinine, and GFR. HEXA is a large-scale cohort designed for the general population to identify environmental genetic factors for major chronic diseases in Koreans. Since 2004, adults 40–70 years of age representing the general population have been recruited from health screenings and medical institutions. The cohort includes data from health checkups and epidemiological surveys as well as follow-up data collected since 2007. Approximately 20 medical institutions exist in Korea nationwide, and approximately 30,000 new participants are enrolled in the study every year. Currently, data from approximately 173,300 adult participants are collected. For the validation of uric acid, we used genetic test results from 3,703 participants in the HEXA cohort study. Validation P values less than 0.05 were considered significant. We grouped significant SNPs by the linkage disequilibrium (LD) and D’ values, and the graphs were generated using Haploview 4.2. software (S1 File, Figure A in S1 File for BUN in chromosome 3, Figure B in S1 File for uric acid in chromosome 4, Figure C in S1 File for uric acid in chromosome 11, Figure D in S1 File for serum creatinine in chromosome 12, Figure E in S1 File for GFR in chromosome 17, Figure F in S1 File for BUN in chromosome 18).

## Results

### Participant characteristics

Clinical and demographic characteristics of the discovery cohort participants are shown in Table 1. A total of 7,064 participants were enrolled in this study. Median age was 51 years old (interquartile range, IQR 44–56), and male was 58.1%. Median GFR was 90.2 (IQR 81.2–100.9) ml/min/1.73m^2^, and, 47 patients (0.67% of whole participants) had advanced kidney disease (GFR below 60 ml/min/1.73m^2^). Genetic information of the whole participants was all included in the discovery samples.

**Table 1:** Demographic and clinical characteristics of participants.

	Whole participants (N = 7,064)
Age (years)	51.0 (44.0–56.0)
Sex (male)	4104 (58.1%)
Weight (Kg)	64.4 (55.2–72.7)
Body mass index	23.0 (21.0–25.0)
Hypertension	1659 (23.6%) (N = 7,034)
Diabetes mellitus	557 (7.9%) (N = 7,063)
Systolic blood pressure (mmHg)	114 (106–125)
Diastolic blood pressure (mmHg)	75 (69–83)
White blood cells (/mm^3^)	5,100 (4,300–6,100)
Hemoglobin (g/dl)	14.4 (13.4–15.4)
Calcium (mg/dl)	9.3 (9.0–9.5)
Phosphorus (mg/dl)	3.5 (3.2–3.8)
Glucose (mg/dl)	95.0 (90.0–103.0)
BUN (mg/dl)	14.0 (12.0–16.0)
Creatinine (mg/dl)	0.82 (0.68–0.94)
GFR (ml/min/1.73m2)	90.2 (81.2–100.9)
Uric acid (mg/dl)	5.4 (4.4–6.4)
Cholesterol (mg/dl)	192.0 (169.0–215.0)
Protein (mg/dl)	7.3 (7.0–7.6)
Albumin (mg/dl)	4.6 (4.4–4.7)
Bilirubin (mg/dl)	0.9 (0.7–1.2)
Alkaline phosphatase (mg/dl)	51.0 (43.0–62.0)
LDL cholesterol (mg/dl)	119.0 (99.0–141.0)
AST (IU/ml)	21.0 (18.0–26.0)
ALT (IU/ml)	16.0 (14.0–27.0)
HbA1c (%)	5.5 (5.4–5.8)

GFR, glomerular filtration rate; LDL, low-density lipoprotein

### Genome-wide association study of BUN, creatinine, GFR, and uric acid

**Fig 1:**
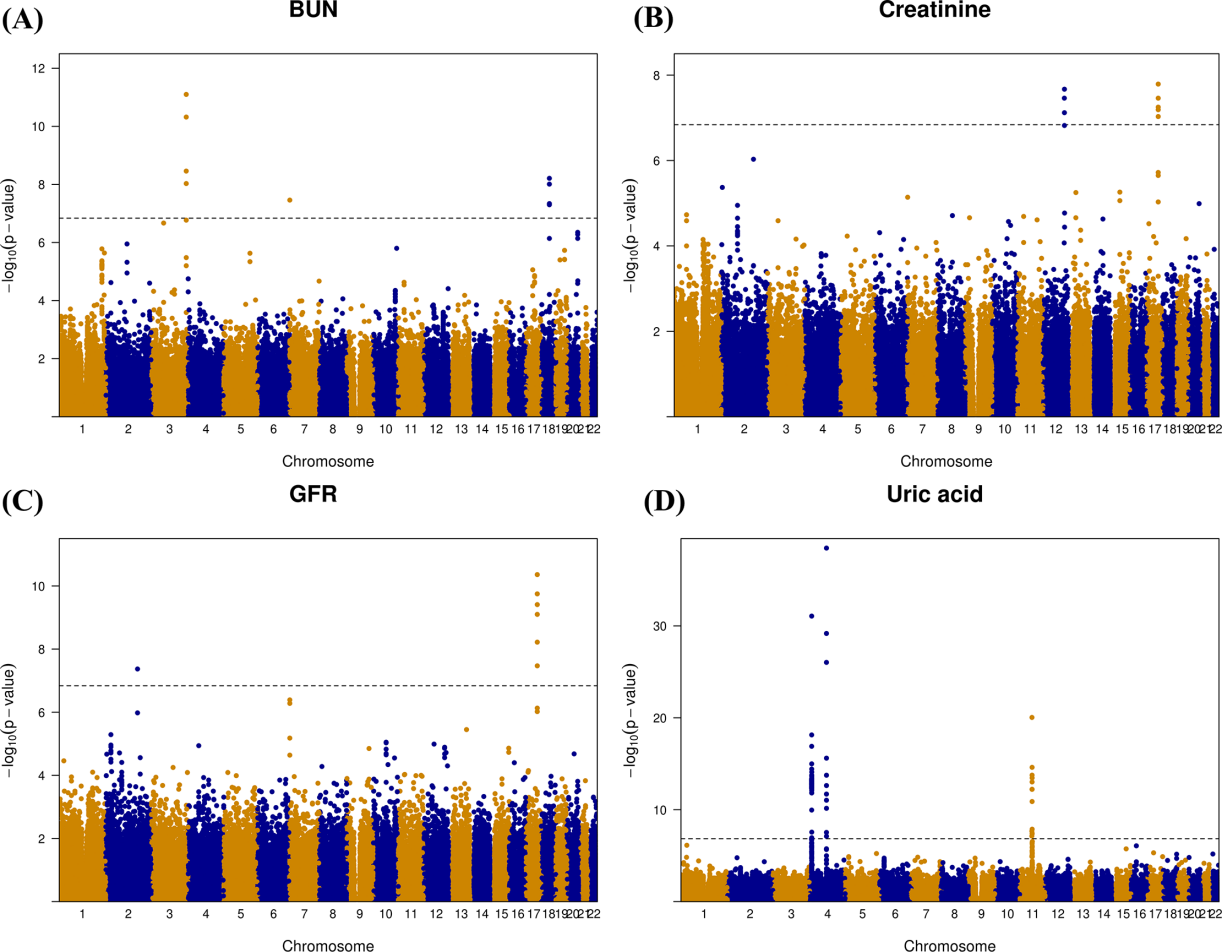
**Genome-wide association analysis -log10 (P-value) vs. genomic position plots (Manhattan plot) for BUN (A), serum creatinine (B), GFR (C), and uric acid (D) in the discovery cohort.** Genetic loci significantly associated (P < 1.45 × 10^−7^ in the discovery set) with BUN were found on chromosomes 3, 7, and 18. Genetic loci significantly associated with serum creatinine were found on chromosomes 12 and 17. Genetic loci significantly associated with GFR were found on chromosomes 2 and 17. Genetic loci significantly associated with uric acid were found on chromosomes 4 and 11.

**Fig 2:**
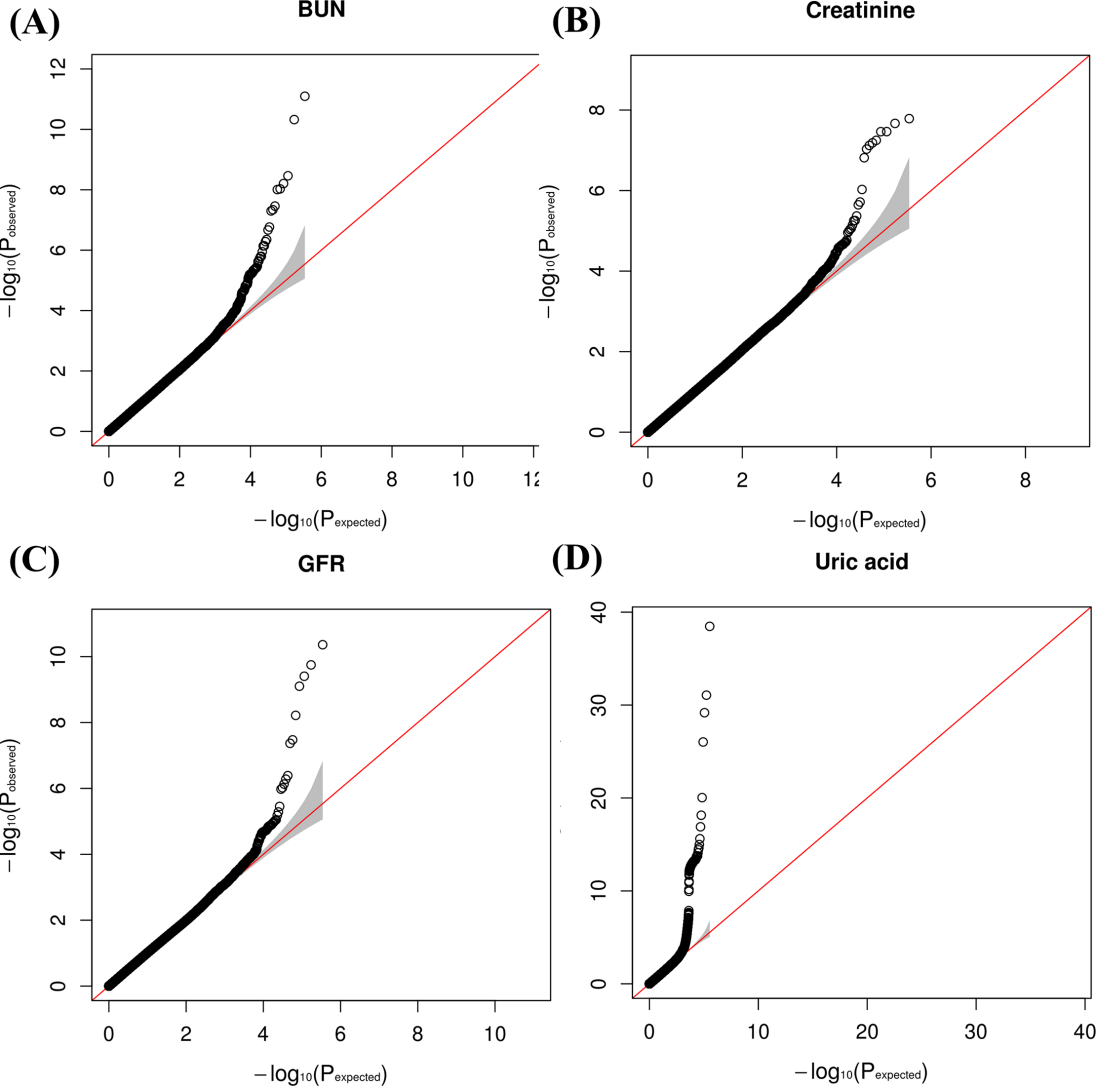
**Quantile-quantile plots of the association test results (expected vs. observed -log10 (P-value)) for BUN (A), serum creatinine (B), GFR (C), and uric acid (D) in the discovery cohort.** The red line shows the expected distribution under the null hypothesis of no association at any locus, and the gray area shows the 95% confidence limits of the null hypothesis distribution.

**Fig 3:**
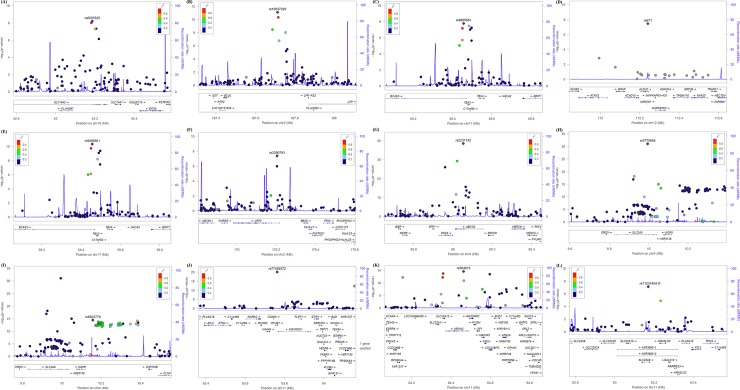
Genetic architecture of genome-wide susceptibility loci significantly associated with kidney disease-related traits in the discovery cohorts. (A) *SLC14A2* on chromosome 18 for BUN. (B): Null intergenic region on chromosome 3 for BUN. (C) *BCAS3* on chromosome 17 for creatinine. (D) *ALDH2* and *HECTD4* on chromosome 12 for creatinine. (E) *BCAS3* on chromosome 17 for GFR. (F) *LRP2* on chromosome 2 for GFR. (G) *ABCG2* on chromosome 4 for uric acid. (H): *SLC2A9* on chromosome 4 for uric acid. (I) intergenic region on chromosome 4 for uric acid. (J) *OTUB1* on chromosome 11 for uric acid. (K) *NRXN2/SLC22A12* on chromosome 11 for uric acid. (L) *SLC22A9* on chromosome 11 for uric acid. The calculated -log10 P values are shown according to genomic position. P values were obtained from the discovery set. Among the SNPs, the most significant SNP with the lowest P value is colored purple. LD values (based on the HapMap CEU sample) are displayed as different colors is marked in colors: red (r2 to top SNP 0.8–0.0), orange (0.4–0.6), green (0.4–0.6), sky blue (0.2–0.4), and dark blue (<0.2). Gene annotations are based on Build 36. The directions of the arrows indicate the direction of transcription.

Results of the genome-wide association study on BUN, creatinine, GFR, and, uric acid across the discovery samples are depicted using a Manhattan plot in Fig 1. The Manhattan plot was drawn using data from the discovery set. Genetic loci significantly associated (P < 1.45 × 10^−7^ in the discovery set) with BUN were found on chromosomes 3, 7, and 18, and those significantly associated with serum creatinine were found on chromosomes 12, and 17. Genetic loci significantly associated with GFR were found on chromosomes 2 and 17. Genetic loci significantly associated with uric acid levels were found on chromosomes 4 and 11. In addition, the distribution statuses of the observed versus expected P values are described using quantile-quantile (QQ) plots in Fig 2. The QQ plots showed good adherence to null expectations. Studies on specific genetic inflation factors did not show substantial inflation of the test statistics on these traits. The calculated values (-log10 P values) are shown according to genomic position using a regional plot chart (Fig 3), and P values were obtained from the discovery set. Among the SNPs, the most significant SNP with the lowest P value is colored purple. On chromosome 18, rs6507625 in *SLC14A2* was identified as the most significant SNP for BUN (P = 6.20 × 10^−9^). On chromosome 3, rs10937329 in an intergenic locus was identified as the most significant SNP for BUN (P = 8.00 × 10^−12^). On chromosome 17, rs9895661 in *BCAS3* was identified as the most significant SNP for creatinine and GFR (P = 1.63 × 10^−8^ for creatinine, P = 4.34 × 10^−11^ for GFR). On chromosome 17, rs757608 in *C17orf82* was also identified as significant SNP for both creatinine and GFR (P = 5.65 × 10^−8^ for creatinine, P = 3.93 × 10^−10^ for GFR). On chromosome 12, rs671 in *ALDH2* was identified as the most significant SNP for creatinine (P = 3.45 × 10^−8^). On chromosome 2, rs2390793 in *LRP2* was identified as the most significant SNP for GFR (P = 4.28 × 10^−8^). On chromosome 4, rs2231142 in *ABCG2* (P = 3.38 × 10^−39^) and rs3775948 in *SLC2A9* (P = 8.59 × 10^−32^) were identified as the most significant SNPs for uric acid. On chromosome 11, rs77459372 in *OTUB1* was identified as the most significant SNP for uric acid (P = 9.12 × 10^−21^).

The most significant SNPs at each genetic locus associated with kidney disease-related traits are summarized in Table 2. We detected 4 SNPs on chromosome 18 (*SLC14A2*), 4 SNPs on chromosome 3 (intergenic loci), and 1 SNP on chromosome 7 (*UNCX*) associated with BUN in the discovery set (Tables 2 and 3). After validation set analysis, 4 SNPs on chromosome 18 (*SLC14A2*) and 4 SNPs on chromosome 3 (intergenic loci) still showed a significant association with BUN. The top SNPs in *SCL14A2* on chromosome 18 and in intergenic loci on chromosome 3 were rs6507625 (*P* = 6.20 × 10^−9^ in the discovery set, *P* = 3.70 × 10^−4^ in the validation set) and rs10937329 (*P* = 8.00 × 10^−12^ in the discovery set, *P* = 2.11 × 10^−9^ in the validation set), respectively. rs11710227 in an intergenic region on chromosome 3 is a newly discovered SNP that showed significant association with BUN.

**Table 2:** Lead SNPs in each genetic loci associated with kidney function-related traits.

	`					Discovery set		Validation set	
Traits	rsIDα	Chromosome	Position (base pair)	Gene	A1	β(SE)	P	β(SE)	P
BUN	rs6507625*	18	43186842	*SLC14A2*	G/A	-0.4115(0.0714)	6.20 × 10^−9^	-0.3318(0.0983)	3.70 × 10^−4^
	rs10937329*	3	187713718	Intergenic	A/T	-0.4044(0.0590)	8.00 × 10^−12^	-0.4695(0.0798)	2.11 × 10^−9^
Creatinine	rs9895661*	17	59456589	*BCAS3*	C/T	0.0121(0.0021)	1.63 × 10^−8^	0.0098(0.0045)	1.38 × 10^−2^
	rs757608*	17	59497277	*C17orf82*	A/G	0.0130(0.0024)	5.65 × 10^−8^	0.0109(0.0043)	5.34 × 10^−3^
	rs671*	12	112241766	*ALDH2*	A/G	0.0158(0.0029)	3.45 × 10^−8^	0.0116(0.0053)	1.41 × 10^−2^
GFR	rs9895661*	17	59456589	*BCAS3*	C/T	-1.705(0.2583)	4.34 × 10^−11^	-0.4283(0.2478)	4.20 × 10^−2^
	rs757608*	17	59497277	*C17orf82/TBX2*	A/G	-1.800(0.2873)	3.93 × 10^−10^	-0.6187(0.2064)	1.37 × 10^−3^
	rs2390793*	2	170205123	*LRP2*	T/C	1.772(0.3231)	4.28 × 10^−8^	0.9836(0.2326)	1.19 × 10^−5^
Uric acid	rs2231142*	4	89052323	*ABCG2/PKD2*	T/G	0.2804(0.0213)	3.38 × 10^−39^	0.2031(0.0286)	7.49 × 10^−13^
	rs3114018*	4	89064581	*ABCG2*	A/C	-0.1648(0.0201)	2.46 × 10^−16^	-0.1548(0.0267)	3.62 × 10^−9^
	rs12511059*	4	89126193	*ABCG2*	T/C	-0.1081(0.0201)	7.84 × 10^−8^	-0.0912(0.0493)	3.22 × 10^−2^
	rs3775948*	4	9995182	*SLC2A9*	G/C	-0.2208(0.0187)	8.59 × 10^−32^	-0.1936(0.0259)	4.55 × 10^−14^
	rs13129697*	4	9926967	*SLC2A9*	G/T	-0.1502(0.0187)	1.03 × 10^−15^	-0.1318(0.0254)	1.14 × 10^−7^
	rs59420943*	4	10384278	Intergenic	T/C	0.1473(0.0189)	8.02 × 10^−15^	0.1153(0.0262)	5.60 × 10^−6^
	rs6839820*	4	10296114	Intergenic	C/T	-0.1424(0.0189)	4.73 × 10^−14^	-0.0942(0.0252)	9.55 × 10^−5^
	rs6823778*	4	10158163	Intergenic	C/T	-0.1481(0.0188)	3.79 × 10^−15^	-0.1061(0.0254)	1.52 × 10^−5^
	rs77459372*	11	63762330	*OTUB1*	A/G	-0.3815(0.0407)	9.12 × 10^−21^	-0.5629(0.209)	3.56 × 10^−3^
	rs55975541*	11	64597201	*CDC42BPG*	A/G	-0.1858(0.0245)	3.49 × 10^−14^	-0.238(0.0633)	8.61 × 10^−5^
	rs504915*	11	64464085	*NRXN2/SLC22A12*	A/T	-0.179(0.0226)	2.45 × 10^−15^	-0.1367(0.0306)	3.96 × 10^−6^
	rs79382056*	11	64154676	*RPS6KA4*	C/T	-0.2361(0.0327)	6.02 × 10^−13^	-0.1565(0.0829)	2.96 × 10^−2^
	rs11231454*	11	63170735	*SLC22A9*	T/C	-0.2221(0.0413)	7.48 × 10^−8^	-0.1035(0.051)	2.12 × 10^−2^
	rs10897526*	11	64559898	*MAP4K2*	T/C	-0.1222(0.0222)	3.67 × 10^−8^	-0.0879(0.036)	7.36 × 10^−3^

*Lead SNP in each genetic loci

**Table 3:** Loci associated with kidney function-related traits (blood urea nitrogen).

`					Discovery Set		Validation Set	
rsIDα	Chromosome	Position (base pair)	Gene	A1/A2	β(SE)	P	β(SE)	P
rs6507625*	18	43186842	*SLC14A2*	G/A	-0.4115(0.0714)	6.20 × 10^−9^	-0.3318(0.0983)	3.70 × 10^−4^
rs1825475	18	43182006	*SLC14A2*	A/G	-0.4106(0.0715)	9.85 × 10^−9^	-0.3339(0.0983)	3.43 × 10^−4^
rs1484873	18	43206985	*SLC14A2*	A/G	-0.3982(0.0728)	4.57 × 10^−8^	-0.3232(0.1057)	1.12 × 10^−3^
rs7232775	18	43202404	*SLC14A2*	C/T	-0.3877(0.0711)	5.02 × 10^−8^	-0.3255(0.0980)	4.52 × 10^−4^
rs10937329*	3	187713718	Intergenic	A/T	-0.4044(0.0590)	8.00 × 10^−12^	-0.4695(0.0798)	2.11 × 10^−9^
rs4686914	3	187717540	Intergenic	T/C	-0.3891(0.0591)	4.74 × 10^−11^	-0.4716(0.0798)	1.78 × 10^−9^
rs16862782	3	187687890	Intergenic	A/C	-0.4411(0.0746)	3.44 × 10^−9^	-0.5377(0.1033)	9.90 × 10^−8^
rs11710227^†^	3	187753995	Intergenic	G/A	-0.3512(0.0611)	9.34 × 10^−9^	-0.3683(0.0844)	6.53 × 10^−6^

*Lead SNP in each genetic loci

^†^Newly discovered SNP.

Five SNPs on chromosome 17 (*BCAS3*, *C17orf82/TBX2*) and 3 SNPs on chromosome 12 (*HECTD4*, *ALDH2*) significantly associated with serum creatinine levels were found in the discovery set. During validation, 4 SNPs (2 on *BCAS3* and 2 on *C17orf82*) on chromosome 17 and 2 SNP on chromosome 12 (*ALDH2/HECTD4*) showed a significant association with serum creatinine levels (Table 4). The lead SNPs in *BCAS3* and *C17orf82* on chromosome 17 and *ALDH2* on chromosome 12 were rs9895661, rs757608, and rs671, respectively.

**Table 4:** Loci associated with kidney function-related traits (serum creatinine).

					Discovery Set		Validation Set	
rsIDα	Chromosome	Position (base pair)	Gene	A1/A2	β(SE)	P	β(SE)	P
rs9895661*	17	59456589	*BCAS3*	C/T	0.0121(0.0021)	1.63 × 10^−8^	0.0098(0.0045)	1.38 × 10^−2^
rs9905274	17	59450441	*BCAS3*	T/C	0.0117(0.0022)	6.52 × 10^−8^	0.0094(0.0047)	2.21 × 10^−2^
rs757608*	17	59497277	*C17orf82*	A/G	0.0130(0.0024)	5.65 × 10^−8^	0.0109(0.0043)	5.34 × 10^−3^
rs9907379	17	59489893	*C17orf82*	T/C	0.0128(0.0024)	9.33 × 10^−8^	0.0101(0.0043)	9.96 × 10^−3^
rs671*	12	112241766	*ALDH2*	A/G	0.0158(0.0029)	3.45 × 10^−8^	0.0116(0.0053)	1.41 × 10^−2^
rs2074356	12	112645401	*HECTD4*	A/G	0.0159(0.0030)	7.58 × 10^−8^	0.0135(0.0053)	5.17 × 10^−3^

*Lead SNP in each genetic loci

Six SNPs on chromosome 17 (*BCAS3*, *C17orf82/TBX2*) and 1 SNP on chromosome 2 (*LRP2*) significantly associated with GFR were found in the discovery and validation sets (Table 5). The lead SNPs in *BCAS3* and *C17orf82/TBX2* on chromosome 17 and *LRP2* on chromosome 2 were rs9895661, rs757608 and rs2390793, respectively. Comparing significant genetic loci associated with serum creatinine levels and GFR showed that 2 genetic loci (*BCAS3* and *C17orf82*) were simultaneously associated with both traits. A genetic locus in *ALDH2* on chromosome 12 was associated with only serum creatinine levels and not with GFR. Genetic loci in *LRP2* on chromosome 2 was associated with only GFR and not with serum creatinine levels.

**Table 5:** Loci associated with kidney function-related traits (glomerular filtration rate).

					Discovery Set		Validation Set	
rsIDα	Chromosome	Position (base pair)	Gene	A1/A2	β(SE)	P	β(SE)	P
rs9895661*	17	59456589	*BCAS3*	C/T	-1.705(0.2583)	4.34 × 10^−11^	-0.4283(0.2478)	4.20 × 10^−2^
rs9905274	17	59450441	*BCAS3*	T/C	-1.656(0.2592)	1.78 × 10^−10^	-0.4848(0.2517)	2.71 × 10^−2^
rs757608*	17	59497277	*C17orf82*	A/G	-1.800(0.2873)	3.93 × 10^−10^	-0.6187(0.2064)	1.37 × 10^−3^
rs9907379	17	59489893	*C17orf82*	T/C	-1.768(0.2872)	7.90 × 10^−10^	-0.5381(0.2090)	5.04 × 10^−3^
rs8068318	17	59483766	*TBX2*	T/C	1.510(0.2593)	6.05 × 10^−9^	0.3816(0.1865)	2.04 × 10^−2^
rs2079795	17	59496649	*C17orf82*	T/C	-1.527(0.2763)	3.35 × 10^−8^	-0.5887(0.1947)	1.25 × 10^−3^
rs2390793*	2	170205123	*LRP2*	T/C	1.772(0.3231)	4.28 × 10^−8^	0.9836(0.2326)	1.19 × 10^−5^

*Lead SNP in each genetic loci

Fourteen genetic loci (3 in *ABCG2/PKD2*, 2 in *SLC2A9*, 3 in intergenic regions on chromosome 4; *OTUB1*, *NRXN2/SLC22A12*, *CDC42BPG*, *RPS6KA4*, *SLC22A9*, and *MAP4K2* on chromosome 11) and 84 SNPs associated with uric acid levels. According to uric acid, 74 SNPs were identified on chromosome 4 (9 in *ABCG2*, 2 in *PKD2*, 7 in *SLC2A9*, 56 in *intergenic region*) and 10 SNPs were identified on chromosome 11 (1 in *OTUB1*, 2 in *NRXN2*, 1 in *CDC42BPG*, 2 in *SLC22A12*, 1 in *RPS6KA4*, 1 in *SLC22A2*, 1 in *MAP4K2*, and 1 in intergenic region) (Table 6). Using linkage disequilibrium (LD) analysis, genetic loci associated with uric acid levels were categorized (Figure B and Figure C in S1 File). On chromosome 4, 8 genetic loci were associated with uric acid levels, and the lead SNPs were rs2231142 in *ABCG2/PKD2* (*P* = 3.38 × 10^−39^); rs3114018 in *ABCG2* (*P* = 2.46 × 10^−16^); rs12511059 in *ABCG2* (*P* = 7.84 × 10^−8^); rs3775948 in *SLC2A9* (*P* = 8.59 × 10^−32^); rs13129697 in *SLC2A9* (*P* = 1.03 × 10^−15^); and rs6839820, rs59420943, and rs6823778 in intergenic regions. On chromosome 11, 6 genetic loci were associated with uric acid levels, and the lead SNPs were rs77459372 in *OTUB1*, rs504915 in *NRXN2/SLC22A12*, rs55975541 in *CDC42BPG*, rs79382056 in *RPS6KA4*, rs11231454 in *SLC22A9*, and rs10897526 in *MAP4K2*.

**Table 6:** Loci associated with kidney function-related traits (uric acid).

					Discovery Set		Validation Set	
rsIDα	Chromosome	Position (base pair)	Gene	A1/A2	β(SE)	P	β(SE)	P
rs2231142*	4	89052323	*ABCG2*	T/G	0.2804(0.0213)	3.38 × 10^−39^	0.2031(0.0286)	7.49 × 10^−13^
rs4148157	4	89020934	*ABCG2*	A/G	0.2527(0.0221)	6.64 × 10^−30^	0.1981(0.0296)	1.26 × 10^−11^
rs2231164	4	89015857	*ABCG2*	C/T	0.1344(0.0191)	1.98 × 10^−12^	0.0832(0.0257)	6.17 × 10^−4^
rs2725220	4	88959922	*PKD2*	C/G	0.2383(0.0222)	9.40 × 10^−27^	0.1831(0.0296)	3.47 × 10^−10^
rs2725201	4	88999306	*PKD2*	T/G	0.1050(0.0189)	2.95 × 10^−8^	0.1558(0.0394)	3.96 × 10^−5^
rs3114018*	4	89064581	*ABCG2*	A/C	-0.1648(0.0201)	2.46 × 10^−16^	-0.1548(0.0267)	3.62 × 10^−9^
rs17731799	4	89068455	*ABCG2*	G/T	-0.1598(0.0208)	1.77 × 10^−14^	-0.1304(0.0277)	1.35 × 10^−6^
rs3114020	4	89083666	*ABCG2*	T/C	-0.1534(0.0209)	2.33 × 10^−13^	-0.1380(0.0278)	3.72 × 10^−7^
rs3109823	4	89064602	*ABCG2*	C/T	-0.1781(0.0261)	9.30 × 10^−12^	-0.1012(0.0337)	1.34 × 10^−3^
rs2622604	4	89078924	*ABCG2*	T/C	-0.1729(0.0265)	7.07 × 10^−11^	-0.0955(0.034)	2.48 × 10^−3^
rs12511059*	4	89126193	*ABCG2*	T/C	-0.1081(0.0201)	7.84 × 10^−8^	-0.0912(0.0493)	3.22 × 10^−2^
rs3775948*	4	9995182	*SLC2A9*	G/C	-0.2208(0.0187)	8.59 × 10^−32^	-0.1936(0.0259)	4.55 × 10^−14^
rs733175	4	10050141	*SLC2A9*	C/T	-0.1502(0.0187)	1.03 × 10^−15^	-0.1318(0.0254)	1.14 × 10^−7^
rs6834555	4	10062326	*SLC2A9*	G/A	-0.1415(0.0187)	4.30 × 10^−14^	-0.129(0.0252)	1.70 × 10^−7^
rs62295971	4	9978142	*SLC2A9*	A/G	0.1216(0.0188)	1.11 × 10^−10^	0.1188(0.0261)	2.65 × 10^−6^
rs13129697*	4	9926967	*SLC2A9*	G/T	-0.1667(0.0187)	7.18 × 10^−19^	-0.1406(0.0259)	3.15 × 10^−8^
rs3733591	4	9922130	*SLC2A9*	C/T	0.1756(0.0205)	1.27 × 10^−17^	0.1545(0.0276)	1.20 × 10^−8^
rs4292327	4	9943700	*SLC2A9*	A/G	0.1991(0.0376)	1.25 × 10^−7^	0.1103(0.0494)	1.28 × 10^−2^
rs59420943*	4	10384278	Intergenic	T/C	0.1473(0.0189)	8.02 × 10^−15^	0.1153(0.0262)	5.60 × 10^−6^
rs9990427	4	10388313	Intergenic	A/G	-0.148(0.0195)	3.63 × 10^−14^	-0.1355(0.0264)	1.44 × 10^−7^
rs11732092	4	10377405	Intergenic	T/G	-0.1402(0.0189)	1.31 × 10^−13^	-0.1037(0.0253)	2.17 × 10^−5^
rs1544599	4	10349168	Intergenic	G/A	-0.1393(0.0188)	1.38 × 10^−13^	-0.0972(0.0253)	6.02 × 10^−5^
rs9990701	4	10388610	Intergenic	A/G	-0.1371(0.0188)	3.55 × 10^−13^	-0.1239(0.0273)	2.93 × 10^−6^
rs6839820*	4	10296114	Intergenic	C/T	-0.1424(0.0189)	4.73 × 10^−14^	-0.0942(0.0252)	9.55 × 10^−5^
rs7670709	4	10288932	Intergenic	C/T	-0.1405(0.0188)	8.53 × 10^−14^	-0.0935(0.0252)	1.06 × 10^−4^
rs6449450	4	10311887	Intergenic	A/G	-0.1462(0.0196)	9.33 × 10^−14^	-0.1365(0.0264)	1.17 × 10^−7^
rs11945358	4	10287559	Intergenic	G/T	-0.1396(0.0188)	1.14 × 10^−13^	-0.0934(0.0252)	1.07 × 10^−4^
rs4697744	4	10298147	Intergenic	A/G	-0.1402(0.0189)	1.20 × 10^−13^	-0.0939(0.0252)	9.82 × 10^−5^
rs6856707	4	10297330	Intergenic	A/G	-0.1386(0.0189)	2.21 × 10^−13^	-0.0942(0.0252)	9.55 × 10^−5^
rs757628	4	10290297	Intergenic	T/C	-0.1387(0.0189)	2.22 × 10^−13^	-0.0944(0.0252)	9.19 × 10^−5^
rs10014800	4	10302493	Intergenic	G/A	-0.1378(0.0189)	3.18 × 10^−13^	-0.0948(0.0253)	9.13 × 10^−5^
rs4698017	4	10298094	Intergenic	G/A	-0.1372(0.0189)	3.71 × 10^−13^	-0.0939(0.0252)	9.88 × 10^−5^
rs10939818	4	10286962	Intergenic	G/T	-0.1362(0.0188)	4.92 × 10^−13^	-0.0889(0.0259)	3.08 × 10^−4^
rs9291683	4	10324160	Intergenic	A/G	0.1119(0.0201)	2.74 × 10^−8^	0.0962(0.027)	1.84 × 10^−4^
rs6823778*	4	10158163	Intergenic	C/T	-0.1481(0.0188)	3.79 × 10^−15^	-0.1061(0.0254)	1.52 × 10^−5^
rs11724092	4	10186604	Intergenic	T/C	-0.1445(0.0188)	1.68 × 10^−14^	-0.0906(0.0253)	1.72 × 10^−4^
rs11723976	4	10186251	Intergenic	T/C	-0.1442(0.0188)	1.88 × 10^−14^	-0.0906(0.0253)	1.72 × 10^−4^
rs4697972	4	10201503	Intergenic	C/A	-0.1435(0.0188)	2.37 × 10^−14^	-0.0904(0.0252)	1.69 × 10^−4^
rs4697973	4	10203152	Intergenic	G/A	-0.1418(0.0188)	4.78 × 10^−14^	-0.0904(0.0252)	1.69 × 10^−4^
rs2159865	4	10193287	Intergenic	T/G	-0.1422(0.0188)	4.84 × 10^−14^	-0.0718(0.0261)	2.97 × 10^−3^
rs55878266	4	10199948	Intergenic	C/T	-0.1418(0.0188)	5.07 × 10^−14^	-0.0902(0.0252)	1.73 × 10^−4^
rs11734623	4	10208303	Intergenic	C/T	-0.1418(0.0188)	5.09 × 10^−14^	-0.0904(0.0252)	1.69 × 10^−4^
rs4697962	4	10188832	Intergenic	G/A	-0.1419(0.0188)	5.57 × 10^−14^	-0.0919(0.0252)	1.33 × 10^−4^
rs2868420	4	10202997	Intergenic	G/A	-0.1415(0.0188)	5.67 × 10^−14^	-0.0904(0.0252)	1.69 × 10^−4^
rs28496435	4	10190318	Intergenic	T/C	-0.1418(0.0188)	5.97 × 10^−14^	-0.0876(0.0253)	2.71 × 10^−4^
rs4697974	4	10205718	Intergenic	A/G	-0.1411(0.0188)	6.35 × 10^−14^	-0.0904(0.0252)	1.71 × 10^−4^
rs2215691	4	10192108	Intergenic	C/T	-0.1414(0.0188)	6.58 × 10^−14^	-0.0875(0.0253)	2.71 × 10^−4^
rs10000104	4	10191766	Intergenic	G/A	-0.1413(0.0188)	6.59 × 10^−14^	-0.0884(0.0253)	2.37 × 10^−4^
rs6836606	4	10198086	Intergenic	G/A	-0.1408(0.0188)	7.10 × 10^−14^	-0.0903(0.0252)	1.70 × 10^−4^
rs6449351	4	10192744	Intergenic	T/C	-0.1408(0.0188)	8.00 × 10^−14^	-0.0851(0.0254)	4.07 × 10^−4^
rs4697963	4	10189483	Intergenic	T/C	-0.1411(0.0189)	8.29 × 10^−14^	-0.0894(0.0253)	2.09 × 10^−4^
rs11726987	4	10190792	Intergenic	C/T	-0.1408(0.0188)	8.64 × 10^−14^	-0.0876(0.0253)	2.71 × 10^−4^
rs6826185	4	10208656	Intergenic	G/A	-0.1396(0.0188)	1.13 × 10^−13^	-0.0905(0.0253)	1.81 × 10^−4^
rs55775442	4	10200204	Intergenic	T/G	-0.1422(0.0191)	1.22 × 10^−13^	-0.0902(0.0252)	1.73 × 10^−4^
rs6832085	4	10194270	Intergenic	T/C	-0.1396(0.0188)	1.26 × 10^−13^	-0.0778(0.0263)	1.55 × 10^−3^
rs11726996	4	10199139	Intergenic	G/T	-0.1411(0.019)	1.32 × 10^−13^	-0.0902(0.0252)	1.73 × 10^−4^
rs7690319	4	10207061	Intergenic	T/C	-0.1394(0.0188)	1.37 × 10^−13^	-0.0903(0.0252)	1.75 × 10^−4^
rs10025980	4	10185799	Intergenic	G/A	-0.1391(0.0188)	1.54 × 10^−13^	-0.0906(0.0253)	1.72 × 10^−4^
rs62285986	4	10189213	Intergenic	A/G	-0.1393(0.0188)	1.57 × 10^−13^	-0.0942(0.0252)	9.37 × 10^−5^
rs4697726	4	10187395	Intergenic	T/C	-0.1399(0.0189)	1.61 × 10^−13^	-0.0836(0.0259)	6.35 × 10^−4^
rs66769576	4	10197663	Intergenic	G/T	-0.139(0.0188)	1.76 × 10^−13^	-0.0902(0.0252)	1.73 × 10^−4^
rs11724760	4	10254162	Intergenic	C/T	-0.1393(0.0189)	1.83 × 10^−13^	-0.0929(0.0253)	1.20 × 10^−4^
rs56391253	4	10187580	Intergenic	G/A	-0.1385(0.0188)	2.04 × 10^−13^	-0.0847(0.0258)	5.15 × 10^−4^
rs4697731	4	10200718	Intergenic	G/A	-0.1388(0.0189)	2.11 × 10^−13^	-0.0904(0.0252)	1.68 × 10^−4^
rs1860895	4	10250779	Intergenic	C/T	-0.1378(0.0188)	2.76 × 10^−13^	-0.0932(0.0253)	1.16 × 10^−4^
rs11735623	4	10251925	Intergenic	G/T	-0.1375(0.0188)	3.01 × 10^−13^	-0.0931(0.0253)	1.18 × 10^−4^
rs10017447	4	10175536	Intergenic	C/A	-0.1378(0.0189)	3.08 × 10^−13^	-0.0976(0.0252)	5.59 × 10^−5^
rs887734	4	10182913	Intergenic	T/C	-0.1371(0.0189)	4.00 × 10^−13^	-0.089(0.0254)	2.27 × 10^−4^
rs4697961	4	10187373	Intergenic	G/A	-0.1375(0.019)	4.39 × 10^−13^	-0.0841(0.0259)	5.89 × 10^−4^
rs1990469	4	10201652	Intergenic	G/T	-0.1383(0.0191)	4.78 × 10^−13^	-0.0904(0.0252)	1.69 × 10^−4^
rs4697950	4	10171644	Intergenic	G/T	-0.1361(0.0188)	5.00 × 10^−13^	-0.099(0.0252)	4.42 × 10^−5^
rs4697724	4	10177818	Intergenic	C/T	-0.1358(0.0189)	6.66 × 10^−13^	-0.0935(0.0252)	1.03 × 10^−4^
rs10026434	4	10208128	Intergenic	C/T	-0.1345(0.0188)	8.27 × 10^−13^	-0.0904(0.0252)	1.69 × 10^−4^
rs9990501	4	10204593	Intergenic	A/G	-0.1318(0.0186)	1.46 × 10^−12^	-0.0896(0.0248)	1.50 × 10^−4^
rs77459372*	11	63762330	*OTUB1*	A/G	-0.3815(0.0407)	9.12 × 10^−21^	-0.5629(0.209)	3.56 × 10^−3^
rs55975541*	11	64597201	*CDC42BPG*	A/G	-0.1858(0.0245)	3.49 × 10^−14^	-0.238(0.0633)	8.61 × 10^−5^
rs504915*	11	64464085	*NRXN2*	A/T	-0.179(0.0226)	2.45 × 10^−15^	-0.1367(0.0306)	3.96 × 10^−6^
rs471618	11	64465403	*NRXN2*	C/T	0.1307(0.0193)	1.29 × 10^−11^	0.1032(0.0257)	2.93 × 10^−5^
rs11231825	11	64360274	*SLC22A12*	C/T	-0.1745(0.0227)	1.75 × 10^−14^	-0.1361(0.0306)	4.43 × 10^−6^
rs9734313	11	64358311	*SLC22A12*	T/C	-0.1654(0.0229)	5.96 × 10^−13^	-0.1365(0.0306)	4.22 × 10^−6^
rs505802	11	64357072	Intergenic	T/C	-0.1689(0.0226)	9.49 × 10^−14^	-0.1364(0.0306)	4.30 × 10^−6^
rs79382056*	11	64154676	*RPS6KA4*	C/T	-0.2361(0.0327)	6.02 × 10^−13^	-0.1565(0.0829)	2.96 × 10^−2^
rs11231454*	11	63170735	*SLC22A9*	T/C	-0.2221(0.0413)	7.48 × 10^−8^	-0.1035(0.051)	2.12 × 10^−2^
rs10897526*	11	64559898	*MAP4K2*	T/C	-0.1222(0.0222)	3.67 × 10^−8^	-0.0879(0.036)	7.36 × 10^−3^

*Lead SNP in each genetic loci

## Discussion

In this study, we used genome-wide association analyses of discovery and validation populations to identify 6 genetic loci (*SLC14A2* on chromosome 18 and intergenic regions on chromosome 3 for BUN; *BCAS3* and *C17orf82* on chromosome 17, and *ALDH2* on chromosome 12 for serum creatinine; *BCAS3* and *C17orf82/TBX2* on chromosome 17, and *LRP2* on chromosome 2 for GFR) that were associated with BUN, serum creatinine, and GFR. rs9895661 in *BCAS3* and rs757608 in *C17orf82* were simultaneously associated with both serum creatinine and GFR. rs11710227 was identified as a newly discovered SNP in an intergenic region on chromosome 3 that showed significant association with BUN. For uric acid, 8 genetic loci on chromosome 4 (2 in *ABCG2*, 1 in *ABCG2/PKD2*, 2 in *SLC2A9*, and 3 in intergenic regions) and 6 genetic loci on chromosome 11 (*OTUB1*, *RPS6KA4*, *NRXN2/SLC22A12*, *CDC42BPG*, *SLC22A9*, *MAP4K2*) were significantly associated with uric acid levels. Among the 345,072 SNPs that met the clustering quality control criteria, 101 SNPs in 20 genetic loci were related to kidney disease-related traits.

rs6507625 in *SLC14A2* on chromosome 18 was the lead SNP associated with BUN. rs6507625 in *SLC14A2* was previously reported to be associated with anthropometric parameters, including BMI and waist circumference, and kidney function-related traits, including serum creatinine and GFR [15]. Other 3 SNPs (rs1825475, rs1484873, and rs7232775) in *SLC14A2* on chromosome 18 were known to be associated with serum creatinine and GFR [15]. rs1484873 and rs7232775 in *SLC14A2* on chromosome 18 were also associated with BUN and hypertension [16,17]. rs10937329 in an intergenic region was the lead SNP on chromosome 3 associated with BUN. rs10937329 was previously proven to be associated with BUN in an analysis of 71,149 Asian populations [18]. In their study, rs10937329 was not related to serum creatinine, GFR, or uric acid levels, and these findings were similar to ours. The SNPs rs11710227, rs16862782, and rs4686914 were identified as novel SNPs on chromosome 3 (intergenic region) that were significantly associated with BUN. Although rs16862782 was reported to be associated with myopia [19], and rs4686914 with metabolic traits [20], the association of these SNP with BUN or other kidney disease-related traits have not been reported. SNPs rs11710227 on chromosome 3 (intergenic region) is a novel SNP that has not been shown to be related to a particular phenotype, and the relationship with BUN was newly revealed in this study.

The SNP rs9895661 in *BCAS3* and SNPs rs757608 in *C17orf82* on chromosome 17 on chromosome 17 were the top SNPs associated with both serum creatinine and GFR. A genetic locus in *BCAS3* on chromosome 17 was previously reported to be associated with serum creatinine and GFR [18,21,22]. Franceschini et al. reported that *BCAS3* was associated with albuminuria [23], and rs9895661 in *BCAS3* was reported to be associated with height [24,25]. The SNP rs9895661 in *BCAS3* was also reported to be associated with CKD [26]. By contrast, Chambers et al. reported that *BCAS3* was not associated with CKD but was associated with serum creatinine levels and GFR [21]. The SNP rs671 in *ALDH2* on chromosome 12 was also associated with serum creatinine levels but not with GFR. A genetic locus in *ALDH2* on chromosome 12 was previously reported to be associated with BUN and serum creatinine but not with GFR [18]. The SNP rs671 in *ALDH2* was reported to be associated with metabolic traits, including diabetes mellitus, and blood pressure [27–29]. The SNP rs671 in *ALDH2* also affects acute rejection after kidney transplantation and drug metabolism in end-stage renal disease patients [30,31]. *C17orf82* was previously reported to be associated with serum creatinine and GFR [21]. The lead SNP rs757608 in *C17orf82* on chromosome 17 was reported to be associated with height [24]. The other SNPs in *C17orf82/TBX2* (rs9907379, rs8068318, and rs2079795) was also reported to be associated with height [24,25,32–34]. In this study, the relationship between the *C17orf82/TBX2* genetic loci on chromosome 17 and GFR has been newly discovered, but this may be due to the relationship between height and GFR. Therefore, in subsequent studies, it is necessary to reconfirm the relevance of GFR by correcting the height factor among other population groups. The SNP rs2390793 in *LRP2* on chromosome 2 was associated with GFR but not with serum creatinine levels. A genetic locus in *LRP2* was previously reported to be associated with BUN, GFR, and proteinuria [21,35,36]. The SNP rs2390793 in *LRP2* was previously reported to be associated with uric acid levels [37].

Uric acid was significantly associated with 84 SNPs in 14 genetic loci on chromosomes 4 (74 SNPs in 8 genetic loci) and 11 (10 SNPs in 6 genetic loci). Five genetic loci (2 in *ABCG2*, 1 in *ABCG2/PKD2*, 2 in *SLC2A9*) and 3 intergenic regions were associated with uric acid on chromosome 4. In *ABCG2* on chromosome 4, 9 SNPs were significant. *ABCG2* (ATP-binding cassette subfamily G member 2) is a protein-coding gene on chromosome 4. Mutations in *ABCG2* are known to be associated with hyperuricemia and the risk of gout [38–40]. The two lead SNPs rs2231142 and rs3114018 in *ABCG2* on chromosome 4 was associated with hyperuricemia or gout [41–50]. In the *PKD2* gene region, SNP rs2725220 and rs2725201 were associated with uric acid levels. *PKD2* is a protein-coding gene at 4q22.1 that encodes a member of the polycystin protein family. Association of the *PKD2* gene with uric acid levels was proven in other previous studies [51]. *SLC2A9* (solute carrier family 2 member 9) is located at 4p16.1 and encodes a member of the SLC2A facilitative glucose transporter family. The *SCL2A9* gene was previously reported to be associated with uric acid levels and CKD progression [52–62]. The two lead SNPs rs3775948 and rs13129697 in *SLC2A9* on chromosome 4 was associated with hyperuricemia or gout [4,41,46,48–50,63–71]. The major 3 SNPs (rs6839820, rs59420943, and rs6823778) on the intergenic region of chromosome 4 were also found to be significantly related to uric acid levels in this study; these findings are novel, as they have not been previously reported.

Six genetic loci (*OTUB1*, *NRXN2/SLC22A12*, *CDC42BPG*, *RPS6KA4*, *SLC22A9*, *and MAP4K2*) were associated with uric acid on chromosome 11. The SNPs rs55975541 in *CDC42BPG* [72], and rs11231825/rs9734313 in *SLC22A12/NRXN2 [46,49,50,72–77],* and rs10897526 in *MAP4K2* [72] on chromosome 4 were previously reported to be associated with hyperuricemia or gout. In general, *OTUB1* is known to be associated with the development and metastasis of colorectal cancer, ovarian cancer, and lung cancer, and increased *OTUB1* expression is associated with worsening prognosis [78–81]. Using a genome-wide association study, Mells et al. reported that a genetic locus in *RPS6KA4* at 11q13 is associated with the development of primary biliary cirrhosis through NF-κB activation [82]. In this study, lead SNPs rs77459372 in *OTUB1*, rs79382056 in *RPS6KA4*, and rs11231454 in *SLC22A9* on chromosome 11 were associated with uric acid levels, which is also a new investigational finding.

Despite the clinical significance of this study, some limitations do exist. Relatively few patients in advanced kidney disease (N = 47, who showed GFR below 60 ml/min/1.73m^2^) were enrolled due to the health screening patients being relatively healthy. In this study, researchers could adjust for only age, sex, diabetes mellitus, hypertension, and BMI as covariates for the analysis of genetic mutations associated with kidney disease-related traits, but many other variables could affect kidney disease-related traits.

In conclusion, we found 20 genetic loci and 101 SNPs that were associated with the kidney disease-related traits serum creatinine, BUN, GFR, and uric acid in the Korean population. SNPs rs11710227 on chromosome 3 (intergenic region) associated with BUN is a novel SNP that has not been reported to be related with specific phenotype. In this study, we also found the six novel genetic loci (3 intergenic region in chromosome 4, and *OTUB1*, *RPS6KA4*, and *SLC22A9* on chromosome 11) associated with uric acid. Studies on genetic mutations have identified genetic risk factors for kidney disease. In addition to clinical findings, such as the degree of proteinuria and kidney biopsy results, results of genetic analysis may be used as risk factors for CKD progression. Because genetic impacts may vary from population to population, additional validation is needed to confirm whether these findings are similar in other populations.

## Supporting information

S1 Table Description of genotyping quality control procedure and analysis workflow. (DOCX)

S2 Table Results of genotyping quality control including minor allele frequency, Hardy-Weinberg equilibrium, and missing rate for blood urea nitrogen. (DOCX)

S3 Table Results of genotyping quality control including minor allele frequency, Hardy-Weinberg equilibrium, and missing rate for serum creatinine. (DOCX)

S4 Table Results of genotyping quality control including minor allele frequency, Hardy-Weinberg equilibrium, and missing rate for glomerular filtration rate. (DOCX)

S5 Table Results of genotyping quality control including minor allele frequency, Hardy-Weinberg equilibrium, and missing rate for uric acid. (DOCX)

S1 File Figures of pairwise linkage disequilibrium on each chromosome. Figure A describes the pairwise linkage disequilibrium for BUN on chromosome 3. Figure B describes the pairwise linkage disequilibrium for uric acid on chromosome 4. Figure C describes the pairwise linkage disequilibrium for uric acid on chromosome 11. Figure D describes the pairwise linkage disequilibrium for serum creatinine on chromosome 12. Figure E describes the pairwise linkage disequilibrium for glomerular filtration rate on chromosome 17. Figure F describes the pairwise linkage disequilibrium for BUN on chromosome 18. The graphs were generated using Haploview 4.2. software. The colors represent D’ values: dark red, high inter-single nucleotide polymorphism (inter-SNP) D’ value; bright red, low inter-SNP D’ value. Linkage disequilibrium blocks are shown.
(ZIP)
