# Supplementary material for: Genome-wide association analysis identifies multiple loci associated with kidney disease-related traits in Korean populations
Source: PLoS One. 2018 Mar 20;13(3):e0194044. doi: 10.1371/journal.pone.0194044 (PMC5860731; doi:10.1371/journal.pone.0194044)
Supplement: S1 Table — (DOCX) [file pone.0194044.s001.docx]

Supplementary Table 1. Description of genotyping quality control procedure and analysis workflow

|  | Participants | SNPs |
| --- | --- | --- |
| Screening | 7,999 | 839,699 |
| Upstream QC |  |  |
| Low DQC value (DQC < 0.82) | -2 |  |
| Low call rate (call rate < 0.97) | -149 |  |
| Plate QC (average call rate < 0.988, plate pass rate < 0.95) | -218 |  |
| Cluster QC (call rate < 0.97, FLD < 3.6, HetSO < -0.1, HomRO < -0.9), |  | -58,080 |
| After Upstream QC | 7,630 | 839,669 |
| Downstream QC |  |  |
| Discordant sex information (0.2 < X-chromsome homozygosity < 0.8) | -8 |  |
| Low call rate (call rate < 0.97) | -31 |  |
| Outlying heterozygosity (heterozygosity rate > mean +/- 3 SD) | -1 |  |
| Related individual (IBS > 0.9) | -288 |  |
| Excessive Missing Genotype (Missing Rate > 0.03) |  | -11,271 |
| Significant Deviation from HWE (HWE test p-value < 10^-5^) |  | -11,821 |
| Low MAF (MAF < 0.05) |  | -413,372 |
| After downstream QC | 7,320 | 345,072 |
| Exclusion of participants who had no laboratory data on kidney disease-related traits | -238 |  |
| Final enrolled participants and target SNPs | 7,064 | 345,072 |

DQC, dish QC; FLD, Fisher’s Linear Discriminant; HWE, Hardy-Weinberg equilibrium; HetSO, Heterozygous Strength Offset; HomRO, Homozygous Ratio Offset; MAF, minor allele frequency; SD, standard deviation; QC, quality control
