# Supplementary material for: Genome-wide association analysis identifies multiple loci associated with kidney disease-related traits in Korean populations
Source: PLoS One. 2018 Mar 20;13(3):e0194044. doi: 10.1371/journal.pone.0194044 (PMC5860731; doi:10.1371/journal.pone.0194044)
Supplement: S2 Table — (DOCX) [file pone.0194044.s002.docx]

S2 Table. Results of genotyping quality control including minor allele frequency, Hardy-Weinberg equilibrium, and missing rate for blood urea nitrogen

| ` |  |  |  |  | D Set |  |  | V Set |  |  |
| --- | --- | --- | --- | --- | --- | --- | --- | --- | --- | --- |
| rsIDα | Chromosome | Position (base pair) | Gene | A1/A2 | MAF | HWE | Missing rate | MAF | HWE | Missing rate |
| rs6507625^*^ | 18 | 43186842 | *SLC14A2* | G/A | 0.1992 | 0.8524 | 0.0031 | 0.1974 | 0.9194 | 0.0014 |
| rs1825475 | 18 | 43182006 | *SLC14A2* | A/G | 0.1978 | 0.9404 | 0.0011 | 0.1970 | 0.8659 | 0.0017 |
| rs1484873 | 18 | 43206985 | *SLC14A2* | A/G | 0.1935 | 0.9087 | 0.0127 | 0.1781 | 0.5719 | 0.0635 |
| rs7232775 | 18 | 43202404 | *SLC14A2* | C/T | 0.2008 | 0.6845 | 0.0008 | 0.1983 | 0.8403 | 0.0000 |
| rs10937329^*^ | 3 | 187713718 | Intergenic | A/T | 0.3752 | 0.4177 | 0.0030 | 0.3738 | 0.2942 | 0.0000 |
| rs4686914 | 3 | 187717540 | Intergenic | T/C | 0.3758 | 0.4477 | 0.0034 | 0.3761 | 0.3873 | 0.0000 |
| rs16862782 | 3 | 187687890 | Intergenic | A/C | 0.1751 | 0.3662 | 0.0017 | 0.1769 | 0.1066 | 0.0000 |
| rs11710227^†^ | 3 | 187753995 | Intergenic | G/A | 0.3244 | 0.4984 | 0.0025 | 0.3135 | 0.9589 | 0.0719 |

^*^Lead SNP in each genetic loci

^†^Newly discovered SNP.
