# Supplementary material for: Genome-wide association analysis identifies multiple loci associated with kidney disease-related traits in Korean populations
Source: PLoS One. 2018 Mar 20;13(3):e0194044. doi: 10.1371/journal.pone.0194044 (PMC5860731; doi:10.1371/journal.pone.0194044)
Supplement: S3 Table — (DOCX) [file pone.0194044.s003.docx]

S3 Table. Results of genotyping quality control including minor allele frequency, Hardy-Weinberg equilibrium, and missing rate for serum creatinine

|  |  |  |  |  | D Set |  |  | V Set |  |  |
| --- | --- | --- | --- | --- | --- | --- | --- | --- | --- | --- |
| rsIDα | Chromosome | Position (base pair) | Gene | A1/A2 | MAF | HWE | Missing rate | MAF | HWE | Missing rate |
| rs9895661^*^ | 17 | 59456589 | *BCAS3* | C/T | 0.4785 | 0.3064 | 0.0027 | 0.4851 | 0.6231 | 0.4538 |
| rs9905274 | 17 | 59450441 | *BCAS3* | T/C | 0.4689 | 0.1461 | 0.0021 | 0.4603 | 0.7003 | 0.4732 |
| rs757608^*^ | 17 | 59497277 | *C17orf82* | A/G | 0.2833 | 0.3206 | 0.0011 | 0.2886 | 0.3012 | 0.0384 |
| rs9907379 | 17 | 59489893 | *C17orf82* | T/C | 0.2839 | 0.4293 | 0.0080 | 0.2825 | 0.4339 | 0.0478 |
| rs671^*^ | 12 | 112241766 | *ALDH2* | A/G | 0.1751 | 0.0172 | 0.0007 | 0.1446 | 0.1522 | 0.0684 |
| rs2074356 | 12 | 112645401 | *HECTD4* | A/G | 0.1602 | 0.0053 | 0.0025 | 0.1730 | 0.509 | 0.0040 |

^*^Lead SNP in each genetic loci
