# Supplementary material for: Genome-wide association analysis identifies multiple loci associated with kidney disease-related traits in Korean populations
Source: PLoS One. 2018 Mar 20;13(3):e0194044. doi: 10.1371/journal.pone.0194044 (PMC5860731; doi:10.1371/journal.pone.0194044)
Supplement: S5 Table — (DOCX) [file pone.0194044.s005.docx]

S5 Table. Results of genotyping quality control including minor allele frequency, Hardy-Weinberg equilibrium, and missing rate for uric acid

|  |  |  |  |  | D Set |  |  | V Set |  |  |
| --- | --- | --- | --- | --- | --- | --- | --- | --- | --- | --- |
| rsIDα | Chromosome | Position  (base pair) | Gene | A1/A2 | MAF | HWE | Missing rate | MAF | HWE | Missing rate |
| rs2231142^*^ | 4 | 89052323 | *ABCG2* | T/G | 0.2663 | 0.5040 | 0.0021 | 0.2733 | 0.1586 | 0.0043 |
| rs4148157 | 4 | 89020934 | *ABCG2* | A/G | 0.2345 | 0.7162 | 0.0018 | 0.2389 | 0.7864 | 0.0000 |
| rs2231164 | 4 | 89015857 | *ABCG2* | C/T | 0.4549 | 0.4877 | 0.0022 | 0.4512 | 0.3171 | 0.0095 |
| rs2725220 | 4 | 88959922 | *PKD2* | C/G | 0.2390 | 1.0000 | 0.0069 | 0.2380 | 0.7857 | 0.0000 |
| rs2725201 | 4 | 88999306 | *PKD2* | T/G | 0.4832 | 0.8117 | 0.0074 | 0.4452 | 0.2064 | 0.5655 |
| rs3114018^*^ | 4 | 89064581 | *ABCG2* | A/C | 0.3274 | 0.8716 | 0.0013 | 0.3285 | 0.5266 | 0.0000 |
| rs17731799 | 4 | 89068455 | *ABCG2* | G/T | 0.3063 | 0.1592 | 0.0184 | 0.3032 | 1.0000 | 0.0249 |
| rs3114020 | 4 | 89083666 | *ABCG2* | T/C | 0.3004 | 0.0176 | 0.0041 | 0.3023 | 1.0000 | 0.0300 |
| rs3109823 | 4 | 89064602 | *ABCG2* | C/T | 0.1602 | 0.0424 | 0.0017 | 0.1627 | 0.0613 | 0.0000 |
| rs2622604 | 4 | 89078924 | *ABCG2* | T/C | 0.1564 | 0.0030 | 0.0001 | 0.1582 | 0.0480 | 0.0000 |
| rs12511059^*^ | 4 | 89126193 | *ABCG2* | T/C | 0.3420 | 0.4587 | 0.0094 | 0.4408 | 0.1417 | 0.7507 |
| rs3775948^*^ | 4 | 9995182 | *SLC2A9* | G/C | 0.4241 | 0.0123 | 0.0028 | 0.4202 | 0.0280 | 0.0043 |
| rs733175 | 4 | 10050141 | *SLC2A9* | C/T | 0.4828 | 0.0345 | 0.0018 | 0.4742 | 0.3065 | 0.0016 |
| rs6834555 | 4 | 10062326 | *SLC2A9* | G/A | 0.4822 | 0.0543 | 0.0017 | 0.4738 | 0.7920 | 0.0008 |
| rs62295971 | 4 | 9978142 | *SLC2A9* | A/G | 0.4482 | 0.1027 | 0.0022 | 0.4523 | 0.0517 | 0.0227 |
| rs13129697^*^ | 4 | 9926967 | *SLC2A9* | G/T | 0.4719 | 0.1465 | 0.0014 | 0.4726 | 0.1091 | 0.0246 |
| rs3733591 | 4 | 9922130 | *SLC2A9* | C/T | 0.2981 | 0.5902 | 0.0011 | 0.3018 | 1.0000 | 0.0141 |
| rs4292327 | 4 | 9943700 | *SLC2A9* | A/G | 0.0680 | 0.7077 | 0.0006 | 0.0715 | 0.3866 | 0.0003 |
| rs59420943^*^ | 4 | 10384278 | Intergenic | T/C | 0.4977 | 0.7568 | 0.0094 | 0.4823 | 0.8915 | 0.0692 |
| rs9990427 | 4 | 10388313 | Intergenic | A/G | 0.3574 | 0.0464 | 0.0038 | 0.3545 | 1.0000 | 0.0070 |
| rs11732092 | 4 | 10377405 | Intergenic | T/G | 0.4788 | 0.6004 | 0.0051 | 0.4757 | 1.0000 | 0.0062 |
| rs1544599 | 4 | 10349168 | Intergenic | G/A | 0.4799 | 0.2743 | 0.0013 | 0.4801 | 0.9737 | 0.0011 |
| rs9990701 | 4 | 10388610 | Intergenic | A/G | 0.4787 | 0.3066 | 0.0022 | 0.4685 | 0.7222 | 0.1394 |
| rs6839820^*^ | 4 | 10296114 | Intergenic | C/T | 0.4817 | 0.3914 | 0.0059 | 0.4794 | 0.8432 | 0.0003 |
| rs7670709 | 4 | 10288932 | Intergenic | C/T | 0.4812 | 0.3071 | 0.0006 | 0.4793 | 0.8175 | 0.0003 |
| rs6449450 | 4 | 10311887 | Intergenic | A/G | 0.3527 | 0.1187 | 0.0028 | 0.3501 | 0.9712 | 0.0000 |
| rs11945358 | 4 | 10287559 | Intergenic | G/T | 0.4811 | 0.1108 | 0.0045 | 0.4794 | 0.7921 | 0.0000 |
| rs4697744 | 4 | 10298147 | Intergenic | A/G | 0.4800 | 0.5056 | 0.0025 | 0.4796 | 0.8176 | 0.0000 |
| rs6856707 | 4 | 10297330 | Intergenic | A/G | 0.4807 | 0.5364 | 0.0031 | 0.4794 | 0.8432 | 0.0003 |
| rs757628 | 4 | 10290297 | Intergenic | T/C | 0.4790 | 0.4324 | 0.0038 | 0.4794 | 0.8432 | 0.0003 |
| rs10014800 | 4 | 10302493 | Intergenic | G/A | 0.4805 | 0.6344 | 0.0028 | 0.4799 | 0.8949 | 0.0046 |
| rs4698017 | 4 | 10298094 | Intergenic | G/A | 0.4818 | 0.5524 | 0.0025 | 0.4797 | 0.8432 | 0.0003 |
| rs10939818 | 4 | 10286962 | Intergenic | G/T | 0.4799 | 0.4187 | 0.0030 | 0.4648 | 0.1488 | 0.0309 |
| rs9291683 | 4 | 10324160 | Intergenic | A/G | 0.3255 | 0.8289 | 0.0013 | 0.3334 | 0.2999 | 0.0003 |
| rs6823778^*^ | 4 | 10158163 | Intergenic | C/T | 0.4939 | 0.3799 | 0.0017 | 0.4904 | 0.7658 | 0.0108 |
| rs11724092 | 4 | 10186604 | Intergenic | T/C | 0.4848 | 0.3185 | 0.0015 | 0.4829 | 0.7159 | 0.0084 |
| rs11723976 | 4 | 10186251 | Intergenic | T/C | 0.4862 | 0.3668 | 0.0022 | 0.4829 | 0.7159 | 0.0084 |
| rs4697972 | 4 | 10201503 | Intergenic | C/A | 0.4757 | 0.0394 | 0.0150 | 0.4758 | 0.7414 | 0.0005 |
| rs4697973 | 4 | 10203152 | Intergenic | G/A | 0.4761 | 0.1991 | 0.0011 | 0.4758 | 0.7414 | 0.0005 |
| rs2159865 | 4 | 10193287 | Intergenic | T/G | 0.4746 | 0.3784 | 0.0028 | 0.4931 | 0.5603 | 0.0769 |
| rs55878266 | 4 | 10199948 | Intergenic | C/T | 0.4759 | 0.2440 | 0.0011 | 0.4760 | 0.6924 | 0.0000 |
| rs11734623 | 4 | 10208303 | Intergenic | C/T | 0.4766 | 0.2159 | 0.0028 | 0.4758 | 0.7415 | 0.0003 |
| rs4697962 | 4 | 10188832 | Intergenic | G/A | 0.4817 | 0.4612 | 0.0022 | 0.4816 | 0.7668 | 0.0003 |
| rs2868420 | 4 | 10202997 | Intergenic | G/A | 0.4760 | 0.2073 | 0.0022 | 0.4758 | 0.7414 | 0.0005 |
| rs28496435 | 4 | 10190318 | Intergenic | T/C | 0.4785 | 0.4053 | 0.0022 | 0.4774 | 1.0000 | 0.0008 |
| rs4697974 | 4 | 10205718 | Intergenic | A/G | 0.4758 | 0.1908 | 0.0015 | 0.4753 | 0.7918 | 0.0008 |
| rs2215691 | 4 | 10192108 | Intergenic | C/T | 0.4789 | 0.5209 | 0.0021 | 0.4770 | 1.0000 | 0.0000 |
| rs10000104 | 4 | 10191766 | Intergenic | G/A | 0.4809 | 0.3794 | 0.0015 | 0.4772 | 1.0000 | 0.0008 |
| rs6836606 | 4 | 10198086 | Intergenic | G/A | 0.4770 | 0.1828 | 0.0024 | 0.4760 | 0.6922 | 0.0003 |
| rs6449351 | 4 | 10192744 | Intergenic | T/C | 0.4719 | 0.2067 | 0.0031 | 0.4741 | 0.9736 | 0.0084 |
| rs4697963 | 4 | 10189483 | Intergenic | T/C | 0.4776 | 0.5359 | 0.0044 | 0.4785 | 0.9209 | 0.0076 |
| rs11726987 | 4 | 10190792 | Intergenic | C/T | 0.4777 | 0.4324 | 0.0030 | 0.4774 | 1.0000 | 0.0008 |
| rs6826185 | 4 | 10208656 | Intergenic | G/A | 0.4761 | 0.1752 | 0.0017 | 0.4777 | 0.7402 | 0.0116 |
| rs55775442 | 4 | 10200204 | Intergenic | T/G | 0.4736 | 0.8472 | 0.0252 | 0.4760 | 0.6924 | 0.0000 |
| rs6832085 | 4 | 10194270 | Intergenic | T/C | 0.4734 | 0.2246 | 0.0028 | 0.4854 | 0.7569 | 0.0837 |
| rs11726996 | 4 | 10199139 | Intergenic | G/T | 0.4819 | 0.5824 | 0.0143 | 0.4760 | 0.6924 | 0.0000 |
| rs7690319 | 4 | 10207061 | Intergenic | T/C | 0.4750 | 0.2158 | 0.0030 | 0.4752 | 0.7664 | 0.0019 |
| rs10025980 | 4 | 10185799 | Intergenic | G/A | 0.4859 | 0.3422 | 0.0015 | 0.4829 | 0.7159 | 0.0084 |
| rs62285986 | 4 | 10189213 | Intergenic | A/G | 0.4784 | 0.4047 | 0.0048 | 0.4808 | 0.8176 | 0.0003 |
| rs4697726 | 4 | 10187395 | Intergenic | T/C | 0.4823 | 0.6678 | 0.0089 | 0.4709 | 0.9463 | 0.0382 |
| rs66769576 | 4 | 10197663 | Intergenic | G/T | 0.4768 | 0.2837 | 0.0052 | 0.4760 | 0.6924 | 0.0000 |
| rs11724760 | 4 | 10254162 | Intergenic | C/T | 0.4776 | 0.6172 | 0.0041 | 0.4775 | 0.9737 | 0.0016 |
| rs56391253 | 4 | 10187580 | Intergenic | G/A | 0.4814 | 0.3666 | 0.0014 | 0.4732 | 0.8665 | 0.0360 |
| rs4697731 | 4 | 10200718 | Intergenic | G/A | 0.4772 | 0.4045 | 0.0048 | 0.4759 | 0.7415 | 0.0003 |
| rs1860895 | 4 | 10250779 | Intergenic | C/T | 0.4788 | 0.3922 | 0.0013 | 0.4776 | 0.9737 | 0.0022 |
| rs11735623 | 4 | 10251925 | Intergenic | G/T | 0.4788 | 0.3924 | 0.0004 | 0.4777 | 0.9737 | 0.0024 |
| rs10017447 | 4 | 10175536 | Intergenic | C/A | 0.4794 | 0.5208 | 0.0027 | 0.4773 | 0.7659 | 0.0062 |
| rs887734 | 4 | 10182913 | Intergenic | T/C | 0.4803 | 0.5209 | 0.0024 | 0.4780 | 0.9472 | 0.0097 |
| rs4697961 | 4 | 10187373 | Intergenic | G/A | 0.4767 | 0.7378 | 0.0129 | 0.4707 | 0.9463 | 0.0384 |
| rs1990469 | 4 | 10201652 | Intergenic | G/T | 0.4706 | 0.9809 | 0.0164 | 0.4758 | 0.7414 | 0.0005 |
| rs4697950 | 4 | 10171644 | Intergenic | G/T | 0.4673 | 0.1818 | 0.0025 | 0.4653 | 0.8168 | 0.0019 |
| rs4697724 | 4 | 10177818 | Intergenic | C/T | 0.4885 | 0.0291 | 0.0216 | 0.4794 | 0.6924 | 0.0003 |
| rs10026434 | 4 | 10208128 | Intergenic | C/T | 0.4722 | 0.1604 | 0.0007 | 0.4758 | 0.7414 | 0.0005 |
| rs9990501 | 4 | 10204593 | Intergenic | A/G | 0.4878 | 0.0005 | 0.0062 | 0.4843 | 0.0147 | 0.0011 |
| rs77459372^*^ | 11 | 63762330 | *OTUB1* | A/G | 0.0582 | 0.0508 | 0.0053 | 0.0040 | 1.0000 | 0.0532 |
| rs55975541^*^ | 11 | 64597201 | *CDC42BPG* | A/G | 0.1813 | 1.0000 | 0.0041 | 0.0618 | 0.1135 | 0.3404 |
| rs504915^*^ | 11 | 64464085 | *NRXN2* | A/T | 0.2296 | 0.3471 | 0.0041 | 0.2289 | 0.0285 | 0.0011 |
| rs471618 | 11 | 64465403 | *NRXN2* | C/T | 0.4349 | 0.2250 | 0.0118 | 0.4266 | 0.6859 | 0.0062 |
| rs11231825 | 11 | 64360274 | *SLC22A12* | C/T | 0.2261 | 0.1859 | 0.0024 | 0.2278 | 0.0249 | 0.0000 |
| rs9734313 | 11 | 64358311 | *SLC22A12* | T/C | 0.2262 | 0.0480 | 0.0134 | 0.2278 | 0.0250 | 0.0003 |
| rs505802 | 11 | 64357072 | Intergenic | T/C | 0.2273 | 0.2951 | 0.0013 | 0.2278 | 0.0279 | 0.0005 |
| rs79382056^*^ | 11 | 64154676 | *RPS6KA4* | C/T | 0.0909 | 1.0000 | 0.0006 | 0.0253 | 0.7254 | 0.0708 |
| rs11231454 | 11 | 63170735 | *SLC22A9* | T/C | 0.0545 | 0.1324 | 0.0014 | 0.0739 | 0.8993 | 0.0929 |
| rs10897526^*^ | 11 | 64559898 | *MAP4K2* | T/C | 0.2414 | 0.4563 | 0.0020 | 0.2311 | 0.5807 | 0.2988 |

^*^Lead SNP in each genetic loci
