# Supplementary figures and images for: Genome-wide association analysis identifies multiple loci associated with kidney disease-related traits in Korean populations
Source: PLoS One. 2018 Mar 20;13(3):e0194044. doi: 10.1371/journal.pone.0194044 (PMC5860731; doi:10.1371/journal.pone.0194044)

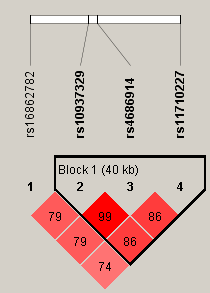

Supplement: S1 File — Figure A describes the pairwise linkage disequilibrium for BUN on chromosome 3. Figure B describes the pairwise linkage disequilibrium for uric acid on chromosome 4. Figure C describes the pairwise linkage disequilibrium for uric acid on chromosome 11. Figure D describes the pairwise linkage disequilibrium for serum creatinine on chromosome 12. Figure E describes the pairwise linkage disequilibrium for glomerular filtration rate on chromosome 17. Figure F describes the pairwise linkage disequilibrium for BUN on chromosome 18. The graphs were generated using Haploview 4.2. software. The colors represent D’ values: dark red, high inter-single nucleotide polymorphism (inter-SNP) D’ value; bright red, low inter-SNP D’ value. Linkage disequilibrium blocks are shown. (ZIP) [file pone.0194044.s006.zip › Figure_A.png]

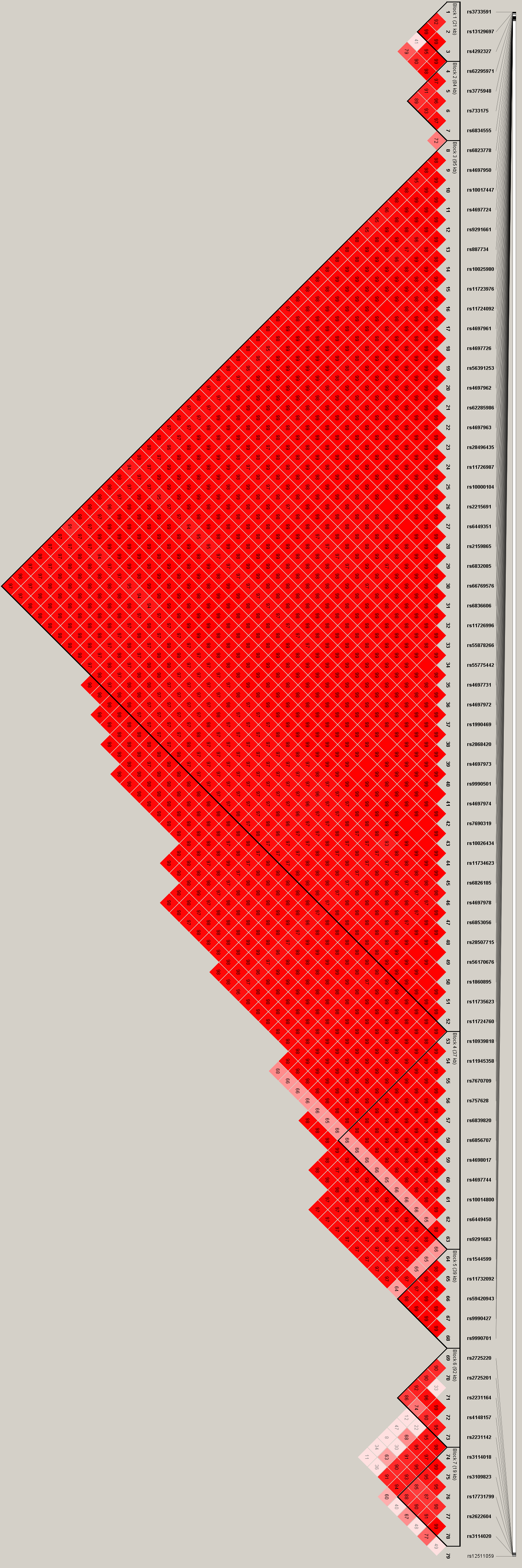

Supplement: S1 File — Figure A describes the pairwise linkage disequilibrium for BUN on chromosome 3. Figure B describes the pairwise linkage disequilibrium for uric acid on chromosome 4. Figure C describes the pairwise linkage disequilibrium for uric acid on chromosome 11. Figure D describes the pairwise linkage disequilibrium for serum creatinine on chromosome 12. Figure E describes the pairwise linkage disequilibrium for glomerular filtration rate on chromosome 17. Figure F describes the pairwise linkage disequilibrium for BUN on chromosome 18. The graphs were generated using Haploview 4.2. software. The colors represent D’ values: dark red, high inter-single nucleotide polymorphism (inter-SNP) D’ value; bright red, low inter-SNP D’ value. Linkage disequilibrium blocks are shown. (ZIP) [file pone.0194044.s006.zip › Figure_B.png]

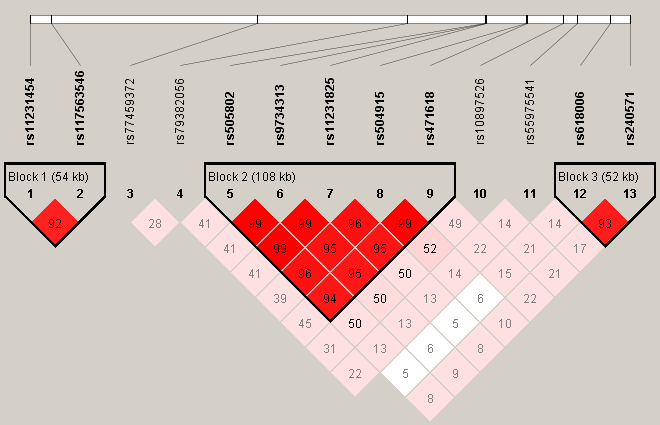

Supplement: S1 File — Figure A describes the pairwise linkage disequilibrium for BUN on chromosome 3. Figure B describes the pairwise linkage disequilibrium for uric acid on chromosome 4. Figure C describes the pairwise linkage disequilibrium for uric acid on chromosome 11. Figure D describes the pairwise linkage disequilibrium for serum creatinine on chromosome 12. Figure E describes the pairwise linkage disequilibrium for glomerular filtration rate on chromosome 17. Figure F describes the pairwise linkage disequilibrium for BUN on chromosome 18. The graphs were generated using Haploview 4.2. software. The colors represent D’ values: dark red, high inter-single nucleotide polymorphism (inter-SNP) D’ value; bright red, low inter-SNP D’ value. Linkage disequilibrium blocks are shown. (ZIP) [file pone.0194044.s006.zip › Figure_C.png]

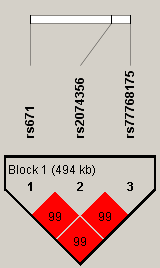

Supplement: S1 File — Figure A describes the pairwise linkage disequilibrium for BUN on chromosome 3. Figure B describes the pairwise linkage disequilibrium for uric acid on chromosome 4. Figure C describes the pairwise linkage disequilibrium for uric acid on chromosome 11. Figure D describes the pairwise linkage disequilibrium for serum creatinine on chromosome 12. Figure E describes the pairwise linkage disequilibrium for glomerular filtration rate on chromosome 17. Figure F describes the pairwise linkage disequilibrium for BUN on chromosome 18. The graphs were generated using Haploview 4.2. software. The colors represent D’ values: dark red, high inter-single nucleotide polymorphism (inter-SNP) D’ value; bright red, low inter-SNP D’ value. Linkage disequilibrium blocks are shown. (ZIP) [file pone.0194044.s006.zip › Figure_D.png]

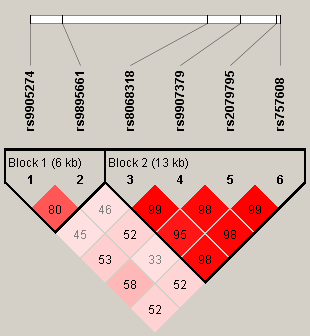

Supplement: S1 File — Figure A describes the pairwise linkage disequilibrium for BUN on chromosome 3. Figure B describes the pairwise linkage disequilibrium for uric acid on chromosome 4. Figure C describes the pairwise linkage disequilibrium for uric acid on chromosome 11. Figure D describes the pairwise linkage disequilibrium for serum creatinine on chromosome 12. Figure E describes the pairwise linkage disequilibrium for glomerular filtration rate on chromosome 17. Figure F describes the pairwise linkage disequilibrium for BUN on chromosome 18. The graphs were generated using Haploview 4.2. software. The colors represent D’ values: dark red, high inter-single nucleotide polymorphism (inter-SNP) D’ value; bright red, low inter-SNP D’ value. Linkage disequilibrium blocks are shown. (ZIP) [file pone.0194044.s006.zip › Figure_E.png]

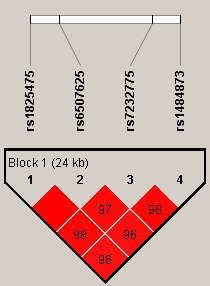

Supplement: S1 File — Figure A describes the pairwise linkage disequilibrium for BUN on chromosome 3. Figure B describes the pairwise linkage disequilibrium for uric acid on chromosome 4. Figure C describes the pairwise linkage disequilibrium for uric acid on chromosome 11. Figure D describes the pairwise linkage disequilibrium for serum creatinine on chromosome 12. Figure E describes the pairwise linkage disequilibrium for glomerular filtration rate on chromosome 17. Figure F describes the pairwise linkage disequilibrium for BUN on chromosome 18. The graphs were generated using Haploview 4.2. software. The colors represent D’ values: dark red, high inter-single nucleotide polymorphism (inter-SNP) D’ value; bright red, low inter-SNP D’ value. Linkage disequilibrium blocks are shown. (ZIP) [file pone.0194044.s006.zip › Figure_F.png]
